# Single cell morphology distinguishes genotype and drug effect in Hereditary Spastic Paraplegia

**DOI:** 10.1038/s41598-021-95995-4

**Published:** 2021-08-17

**Authors:** Gautam Wali, Shlomo Berkovsky, Daniel R. Whiten, Alan Mackay-Sim, Carolyn M. Sue

**Affiliations:** 1 Department of Neurogenetics, Kolling Institute of Medical Research, The University of Sydney, Sydney, NSW 2065 Australia; 2 Centre for Health Informatics, Macquarie University, Sydney, Australia; 3 Griffith Institute for Drug Discovery, Griffith University, Brisbane, Australia

**Keywords:** Neurological disorders, Fluorescence imaging

## Abstract

A central need for neurodegenerative diseases is to find curative drugs for the many clinical subtypes, the causative gene for most cases being unknown. This requires the classification of disease cases at the genetic and cellular level, an understanding of disease aetiology in the subtypes and the development of phenotypic assays for high throughput screening of large compound libraries. Herein we describe a method that facilitates these requirements based on cell morphology that is being increasingly used as a readout defining cell state. In patient-derived fibroblasts we quantified 124 morphological features in 100,000 cells from 15 people with two genotypes (*SPAST* and *SPG7*) of Hereditary Spastic Paraplegia (HSP) and matched controls. Using machine learning analysis, we distinguished between each genotype and separated them from controls. Cell morphologies changed with treatment with noscapine, a tubulin-binding drug, in a genotype-dependent manner, revealing a novel effect on one of the genotypes (*SPG7*). These findings demonstrate a method for morphological profiling in fibroblasts, an accessible non-neural cell, to classify and distinguish between clinical subtypes of neurodegenerative diseases, for drug discovery, and potentially for biomarkers of disease severity and progression.

## Introduction

Neurodegenerative diseases typically are associated with genetic mutations. These mutations alter cellular processes, triggering a cascade of downstream events eventuating in cell dysfunctions or death, thereby leading to clinical phenotype. Studies show multiple cell dysfunctions in patient-derived cells from people with different neurodegenerative disease^1–5^. For classification of disease subtypes, it would be useful to use a composite readout of multiple cell functions that can be used across disease genotypes, without initially depending on an understanding of disease aetiology. Such a readout is cell morphology, which is strongly linked to pathology. For example, basic measures of cell and nucleus morphology (area, perimeter, long axis, short axis, aspect ratio) as well as features like protrusions (total number, mean length, primary protrusion number, secondary protrusion number, ratio of secondary to primary protrusions) and other derived features were used to predict metastatic potential in breast cancer cells^6^. “Cell morphology” can also include morphology of cell components such as mitochondria. Classification of idiopathic Parkinson’s disease cells was improved by quantifying cell and mitochondrial features. Although there was extensive overlap in the distributions of each feature between groups, machine learning using all the features classified idiopathic Parkinson’s disease from controls with a predictive power of 0.87 (the area under the receiving operating characteristic curve)^7^. Interestingly, fibroblasts from people with Parkinson’s disease with *LRRK2* mutations have different mitochondrial morphology even though they are clinically indistinguishable from those with idiopathic Parkinson’s disease^8^. This illustrates the potential for distinguishing between disease genotypes that are not evident in clinical phenotypes. Additionally, morphology is linked to a wide range of cell functions such as altered cytoskeletal dynamics^9^, altered mitochondrial function^10^, apoptosis^11^ and many more.

To date, studies of morphology of single cells or their components are usually confined to quantification of separate features (for example mitochondrial aspect ratio measuring mitochondrial length) to distinguish between disease and control cells^12^. Combining multiple features greatly improves cell classification. In this study we used morphometry to quantify multiple morphological features of cell and nucleus shape and size, mitochondrial morphology, and microtubule structure in cells derived from people with HSP due to two genotypes (*SPAST* and *SPG7*) comparing them to cells from age-matched healthy controls. We tested the efficacy of machine-learning based classification of cells using individual cell morphological features. We tested the effects on cell morphology of treatment with noscapine, a tubulin-binding drug that is therapeutic in *SPAST* HSP cells^13^ but not expected to affect *SPG7* HSP cells due to difference in genotype. We used patient-derived fibroblast as an easily accessible cell available in all patients with neurodegenerative diseases.

**Figure 1:**
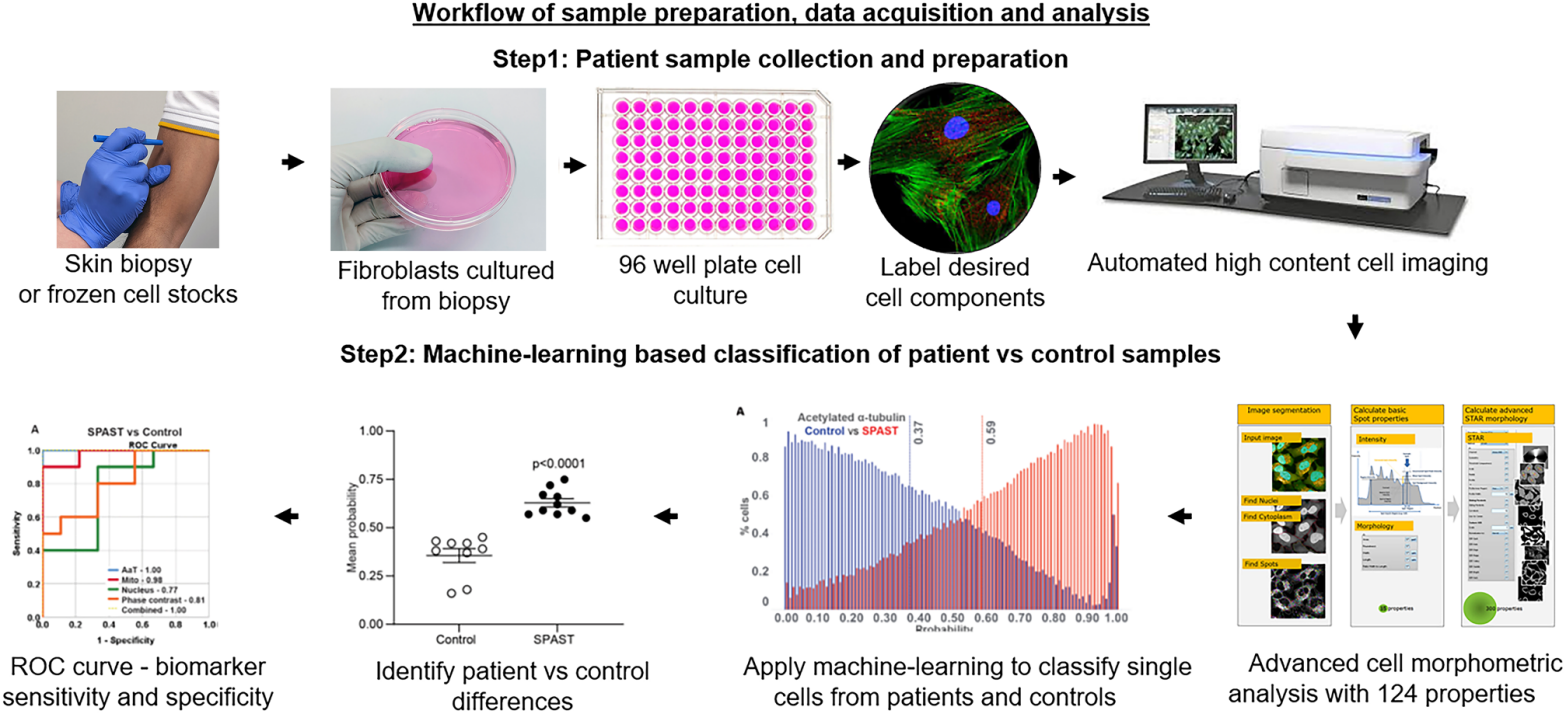
Overview of the workflow of the cellular morphology phenotypic biomarker development approach.

We describe the use of automated image acquisition to capture large numbers of images of individual cells (thousands per group) and the quantification of morphological features of cells and different cell components (124 features per cell based on various measures of size, shape, texture, intensity, distribution pattern, Supplementary Fig. 1) to produce a rich profile of each cell. For each experiment, millions of morphological feature values are generated per disease/control group. The application of machine-learning-based logistical regression allows the classification of disease cases from controls and the evaluation of effects following pharmacological treatment (process outlined in Fig. 1).

## Results

### Machine learning improves detection of drug effects on cell morphology

**Figure 2:**
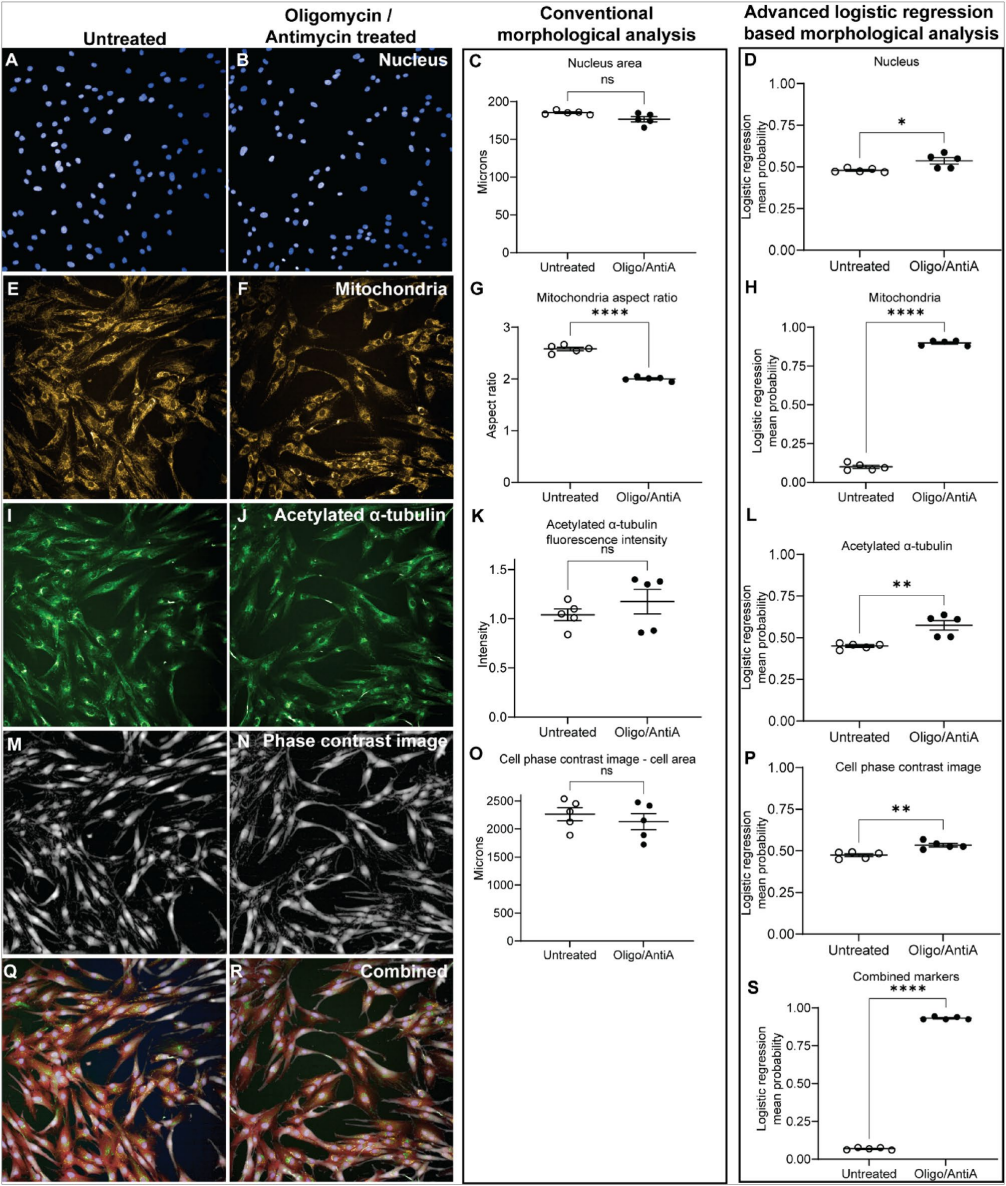
Conventional vs advanced image analysis approaches. We compared the two approaches using images of cells treated with mitochondria respirator chain inhibitors Oligomycin and AntimycinA. Images of cells labelled to identify cell nucleus (**A**, **B**), mitochondria (**E**, **F**), acetylated α-tubulin (**I**, **J**), label-free phase contrast cell images (**M**, **N**) and the combination of all markers (**Q**, **R**) were analysed. Conventional analysis identified differences in mitochondria morphology between the two cell groups (1.25 fold difference) (**G**) and did not identify any differences with the other markers (**C**, **K**, **O**). In contrast, the advanced image analysis approach identified an amplified mitochondria morphology difference of 9.80-fold between the two groups (**H**) and identified significant morphological differences in cell components: nucleus (**D**), acetylated α-tubulin (**L**), label-free phase contrast images (**P**) and the combination of all markers (**S**). Mean values were compared using students t-test.

We assessed the effects of mitochondria function inhibitors on the cell and cell component morphologies: nucleus (Hoechst, Fig. 2A–D), mitochondria (TOM20, Fig. 2E–H), stable microtubules (acetylated α-tubulin, Fig. 2I–L) and cell morphology (phase contrast images, Fig. 2M–P). Conventional morphological analysis (such as mitochondrial aspect ratio) identified significant group differences due to the drug treatment only in mitochondria (p < 0.0001, Fig. 2G). In contrast, there were statistically significant group differences in all measures (Fig. 2D,H,L,P) when we measured a diverse range of morphological features on the same images (31 features per cell or cell component, Supplementary Fig. 1). Using the morphological features, machine-learning based logistical regression analysis was applied to compare the untreated and treated cells. For logistic regression analysis, untreated cells were coded as 0 and treated patient cells as 1. Comparison of the mitochondria morphology of untreated and treated cells demonstrated larger effect size with the machine learning based analysis compared to conventional analysis (Fig. 2H, 9.8-fold difference between untreated and treated cells; Fig. 2G, 1.25-fold difference). This difference is further amplified when all markers are combined (Fig. 2Q,R,S, 13.4-fold difference between untreated and treated cells).

### Machine learning-based cell morphological analysis distinguishes HSP genotypes

#### SPAST vs control

**Figure 3:**
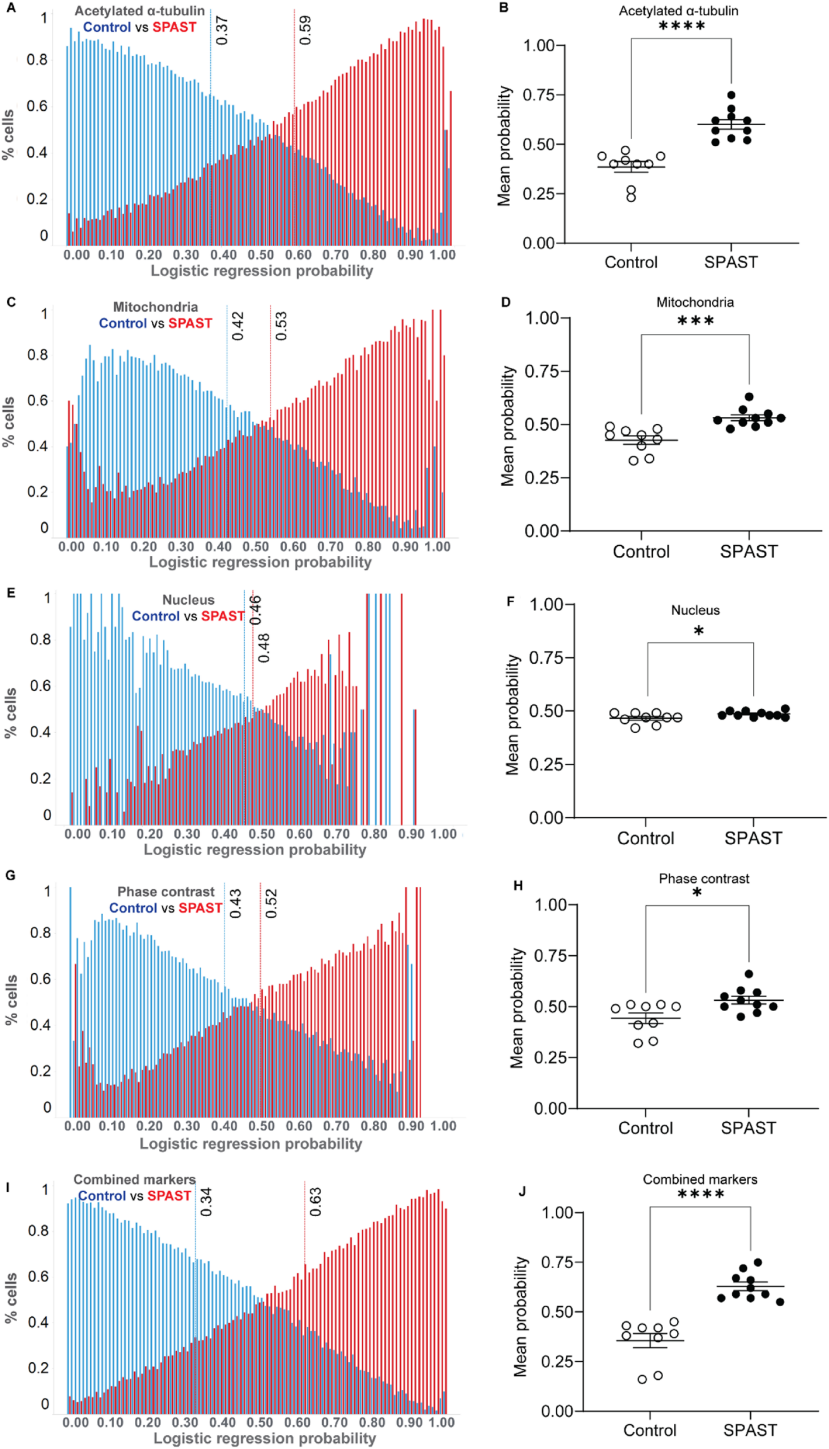
Logistic regression analysis of *SPAST* patient vs control samples. Histogram of single cell logistic regression probability scores is presented for 71,942 cells from 10 *SPAST* patient and 9 control individuals for multiple cell components: acetylated α-tubulin (**A**), mitochondria (**C**), nucleus (**E**), cell phase contrast (**G**) and combined markers (**I**). Dotted lines indicate mean probability score values. The mean logistic regression probability score showing all the individual patient and control cell lines is presented for acetylated α-tubulin (**B**), mitochondria (**D**), nucleus (**F**), the cell phase contrast image (**H**) and combined markers (**J**). Mean values were compared using students t-test.

Logistic regression analysis of 8,920,808 morphological feature values from 71,942 cells (124 features per cell) of 10 *SPAST* patient and 9 healthy control individuals showed significant differences between the two groups (Fig. 3). For logistic regression analysis, control cells were coded as 0 and *SPAST* patient cells as 1. Histogram of the probability scores for all 71,942 cells from both groups are shown for all markers: acetylated α-tubulin (Fig. 3A), mitochondria (Fig. 3C), nucleus (Fig. 3E), cell phase contrast (Fig. 3G) and combined markers (Fig. 3I). The red and blue dotted lines show the mean probability scores of all the *SPAST* patient cells (from 10 individuals) and controls (from 9 individuals) respectively.

For group comparisons, we compared the mean probability scores for all individuals between the *SPAST* and control groups for all markers. The mean logistic regression probability scores were significantly different between *SPAST* and control groups for all markers: acetylated α-tubulin (Fig. 3B, *SPAST* mean: 0.59, control mean: 0.37, p < 0.0001), mitochondria (Fig. 3D, *SPAST* mean: 0.53, control mean: 0.42, p = 0.0003), nucleus (Fig. 3F, *SPAST* mean: 0.48, control mean: 0.46, p = 0.0407), the cell (Fig. 3H, *SPAST* mean: 0.52, control mean: 0.43, p = 0.0123) and combined markers (Fig. 3J, *SPAST* mean: 0.63, control mean: 0.34, p < 0.0001), showed statistically significant *SPAST* vs control differences. The probability scores of individual patients and controls for acetylated α-tubulin (Fig. 3B) and the combined markers (Fig. 3J) did not overlap between the two groups, indicating that they were the most effective markers in distinguishing the two groups.

#### SPG7 vs control

**Figure 4:**
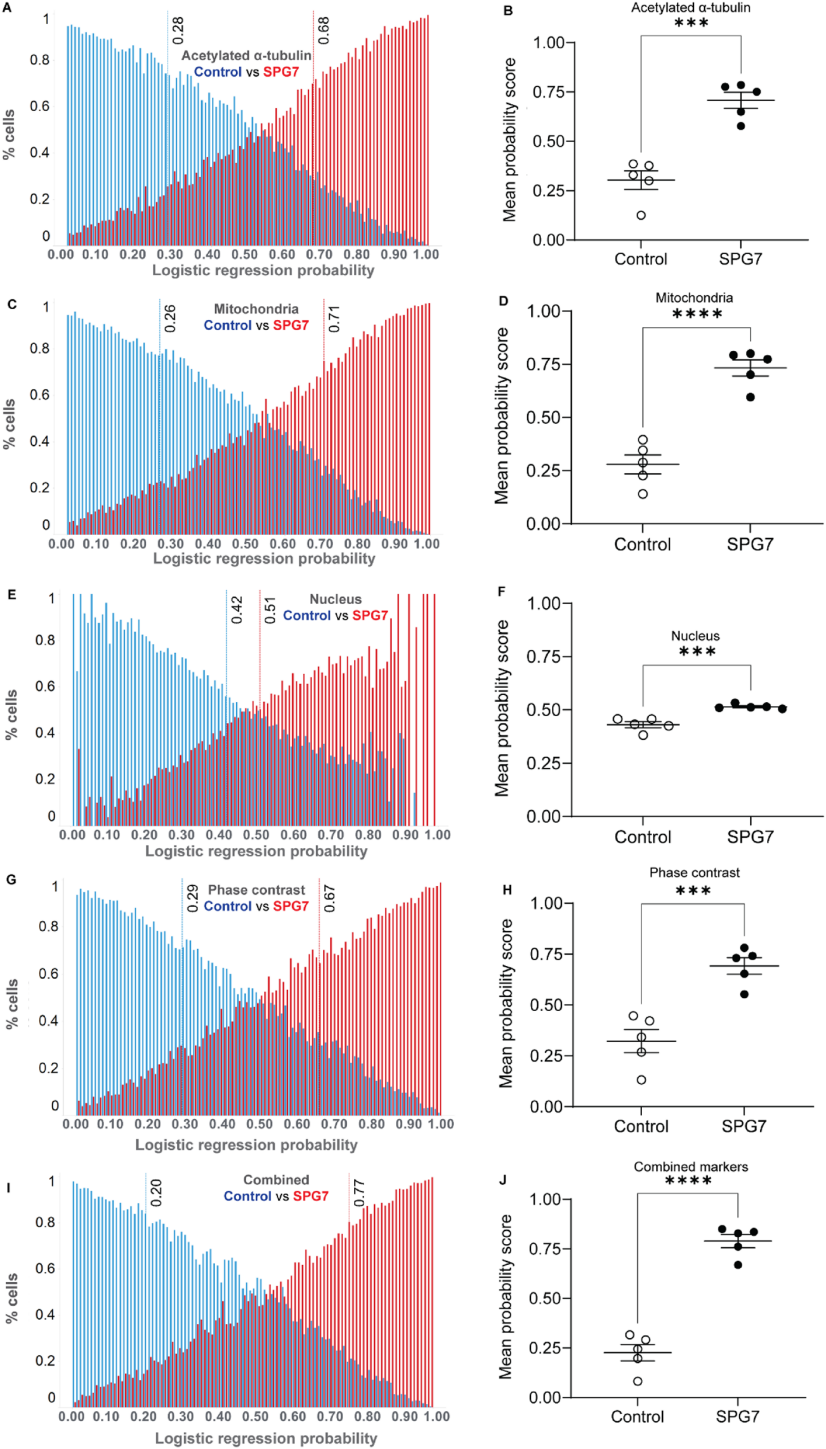
Logistic regression analysis of *SPG7* patient vs control samples. Histogram of single cell logistic regression probability scores is presented for 32,268 cells from 5 *SPG7* patient and 5 control individuals for multiple cell components: acetylated α-tubulin (**A**), mitochondria (**C**), nucleus (**E**), cell phase contrast (**G**) and combined markers (**I**). Dotted lines indicate mean probability score values. The mean logistic regression probability score showing all the individual patient and control cell lines is presented for acetylated α-tubulin (**B**), mitochondria (**D**), nucleus (**F**), the cell phase contrast image (**H**) and combined markers (**J**). Mean values were compared using students t-test.

Logistic regression analysis of 4,001,232 morphological feature values from 32,268 cells (124 features per cell) of 5 *SPG7* patients and 5 healthy controls showed significant differences between the two groups (Fig. 4). For logistic regression analysis, control cells were coded as 0 and *SPG7* patient cells as 1. Histogram of the probability scores for all 32,268 cells from both groups are shown for all markers: acetylated α-tubulin (Fig. 4A), mitochondria (Fig. 4C), nucleus (Fig. 4E), cell phase contrast image (Fig. 4G) and combined markers (Fig. 4I). The red and blue dotted lines show the mean probability scores of all the *SPG7* patient cells (from 5 individuals) and controls (from 5 individuals) respectively.

For group comparisons, we compared the mean probability scores for all individuals between the *SPG7* and control groups for all markers. The mean logistic regression probability scores were significantly different between *SPG7* and control groups for all markers: acetylated α-tubulin (Fig. 4B, *SPG7* mean: 0.68, control mean: 0.28, p = 0.0002), mitochondria (Fig. 4D, *SPG7* mean: 0.71, control mean: 0.26, p < 0.0001), the nucleus (Fig. 4F, *SPG7* mean: 0.51, control mean: 0.42, p = 0.0005), and the cell phase contrast image (Fig. 4H, *SPG7* mean: 0.67, control mean: 0.29, p = 0.0007) and all markers combined (Fig. 4J, *SPG7* mean: 0.77, control mean: 0.20, p < 0.0001). For all markers, the individual values did not overlap, showing that all markers were effective in distinguishing individuals of the two groups.

#### SPAST vs SPG7

**Figure 5:**
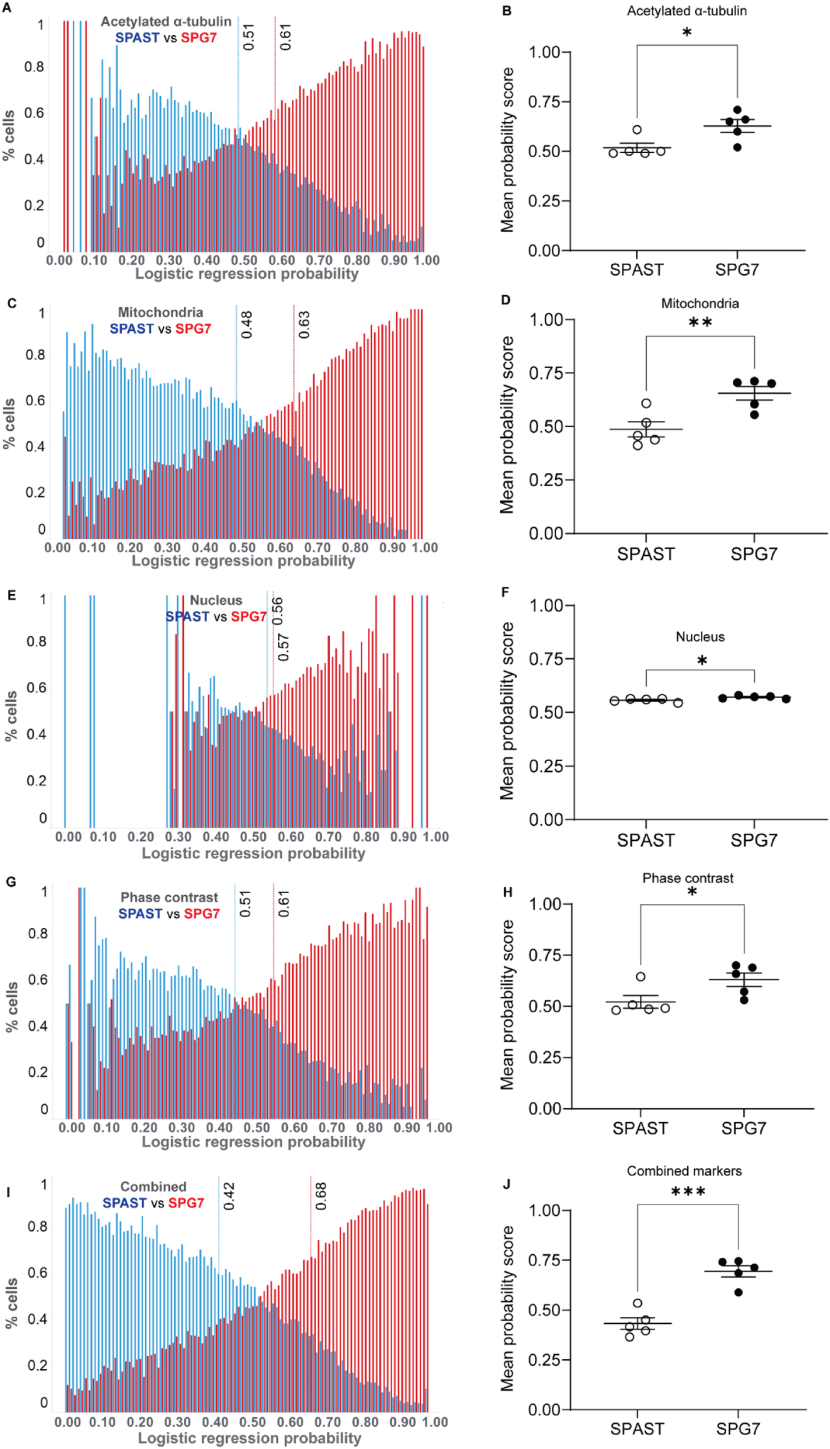
Logistic regression analysis of *SPAST* patient vs SPG7 patient samples. Histogram of single cell logistic regression probability scores is presented for 26,525 cells from 5 *SPG7* patient and 5 control individuals for multiple cell components: acetylated α-tubulin (**A**), mitochondria (**C**), nucleus (**E**), cell phase contrast (**G**) and combined markers (**I**). Dotted lines indicate mean probability score values. The mean logistic regression probability score showing all the individual patient and control cell lines is presented for acetylated α-tubulin (**B**), mitochondria (**D**), nucleus (**F**), the cell (phase contrast image) (**H**) and combined markers (**J**). Mean values were compared using students t-test.

Logistic regression analysis of 3,289,100 morphological feature values from 26,525 cells (124 features per cell) of 5 *SPG7* and 5 *SPAST* patients showed significant differences between the two groups (Fig. 5). Histogram of probability scores for all 26,525 cells from both groups are shown for all markers: acetylated α-tubulin (Fig. 5A), mitochondria (Fig. 5C), nucleus (Fig. 5E), cell phase contrast (Fig. 5G) and combined markers (Fig. 5I). The red and blue dotted lines show the mean probability scores of all the *SPG7* patient cells (from 5 individuals) and *SPAST* (from 5 individuals) respectively.

For group comparisons, we compared the mean probability scores for all individuals between the *SPAST* and *SPG7* groups for all markers. The mean logistic regression probability scores were significantly different between *SPAST* and *SPG7* groups for all markers: acetylated α-tubulin (Fig. 5B, *SPAST* mean: 0.51, *SPG7* mean: 0.61, p = 0.0240), mitochondria (Fig. 5D, *SPAST* mean: 0.48, *SPG7* mean: 0.63, p = 0.0074), nucleus (Fig. 5F, *SPAST* mean: 0.56, *SPG7* mean: 0.57, p = 0.0173), the cell phase contrast image (Fig. 5H, *SPAST* mean: 0.51, *SPG7* mean: 0.61, p = 0.0455) and all markers combined (Fig. 5J, *SPAST* mean: 0.42, *SPG7* mean: 0.68, p = 0.0002), showed significant *SPAST-SPG7* differences. Although the analysis distinguished *SPAST-SPG7* patient samples for all markers, some individual values overlapped between the two groups for each marker except the combined set of morphological features.

#### Sensitivity and specificity of detecting genotype differences in cell morphology

Receiver operating curve (ROC) analysis plots sensitivity against 1-specificity using true positive rates and false positive rates of classification. The area under the curve (AUC) is an indicator of the strength of the classification from 1 (100% sensitivity and 100% specificity) to 0, where 0.5 represents no classification seen in randomly selected samples.

**Figure 6:**
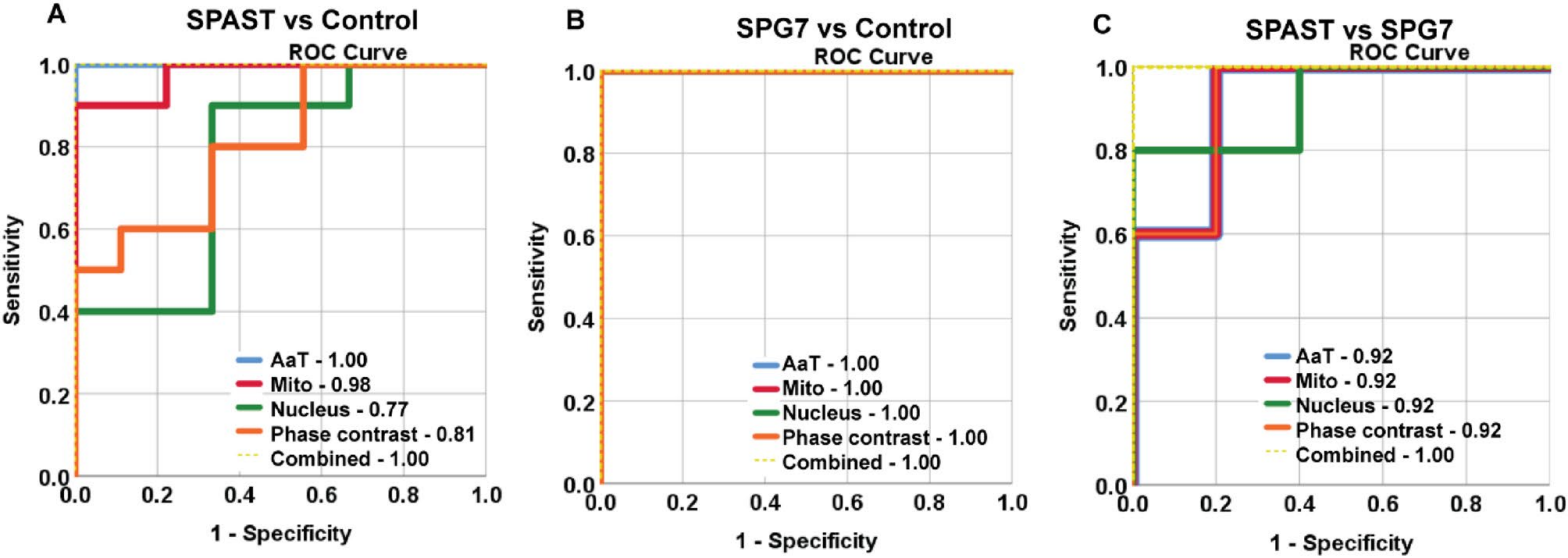
ROC curve analysis. ROC curve analysis of (**A**) *SPAST* vs control samples (**B**) *SPG7* vs control samples (**C**) *SPAST* vs *SPG7* samples. The AUC for all markers for *SPG7* vs control samples is 1.00 and hence all lines overlap and cannot be distinguished. AUC is mentioned for all markers. *AaT* Acetylated α-tubulin, *Mito* Mitochondria, Nucleus, Cell phase contrast and Combined: All markers combined.

ROC analysis of the *SPAST* vs control classification showed 100% specificity and 100% sensitivity for the combined set of morphological features (AUC = 1.00, Fig. 6A). Subsets of features also classified the cases and controls with varying degrees sensitivity and specificity: acetylated α-tubulin, AUC = 1.00; mitochondria, AUC = 0.98; nucleus, AUC = 0.77; cell phase contrast, AUC = 0.81 (Fig. 6A).

ROC analysis of the *SPG7* vs control classification showed 100% specificity and 100% sensitivity for the combined set of morphological features (AUC = 1.00) as well as all the subsets of markers: acetylated α-tubulin, AUC = 1.00; mitochondria, AUC = 1.00; nucleus, AUC = 1.00; cell phase contrast, AUC = 1.00 (Fig. 6B).

ROC analysis of the *SPAST* vs SPG7 classification showed 100% specificity and 100% sensitivity for the combined set of morphological features (AUC = 1.00). Subsets of features consistently classified the cases with controls, AUCs: acetylated α-tubulin, AUC = 0.92; mitochondria, AUC = 0.92; nucleus, AUC = 0.92; cell phase contrast, AUC = 0.92 (Fig. 6C).

### Genotype differences in cell morphology after drug treatment

#### SPAST

The logistic regression model built while analysing control and *SPAST* patient samples (Fig. 3) was used to test if the morphology of noscapine treated *SPAST* patient cells are more similar to untreated control or untreated *SPAST* patient cells. We applied the model to test noscapine treated *SPAST* patient cells (33,764 cells from 10 individuals) and to untreated control and *SPAST* patient cells presented above in the *SPAST* vs controls section (Fig. 3) for comparison.

**Figure 7:**
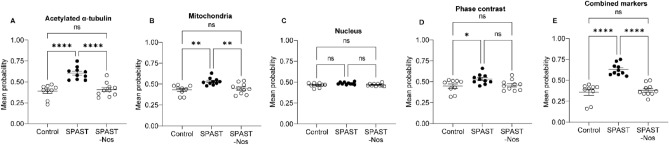
Logistic regression analysis of noscapine-treated *SPAST* patient cells. We compared noscapine treated *SPAST* patient cells (33,764 cells from 10 individuals) to untreated control and *SPAST* patient cells presented above in the *SPAST* vs controls section (Fig. 3) for comparison. Noscapine-treated *SPAST* patient cells had logistic regression probability values comparable to controls for all markers: acetylated α-tubulin (**A**), mitochondria (**B**), nucleus (**C**), phase contrast (**D**) and all markers combined (**E**). Mean values were compared using one way ANOVA.

Noscapine treatment altered morphologies of all markers of *SPAST* patient cells to varying degrees (Fig. 7). The logistic regression probability mean values for all markers were significantly different among the untreated control, untreated *SPAST* and noscapine treated *SPAST* patient groups (ANOVA analysis: acetylated α-tubulin p < 0.0001, mitochondria p = 0.0008, nucleus p = 0.0477, cell phase contrast p = 0.0210, markers combined p < 0.0001).

Noscapine treatment rescued *SPAST* patient cell acetylated α-tubulin and mitochondria morphologies. This effect was also observed for the combined markers. ANOVA Tukey’s multiple comparison test indicated that noscapine treated *SPAST* patient cells were different to untreated *SPAST* patient cells and similar to untreated control cells based on acetylated α-tubulin, mitochondria and combined marker morphologies. Acetylated α-tubulin (Fig. 7A, *SPAST*-Nos vs untreated controls p = 0.8469; *SPAST*-Nos vs untreated *SPAST* p < 0.0001); mitochondria (Fig. 7B, *SPAST*-Nos vs untreated controls p = 0.8045; *SPAST*-Nos vs untreated *SPAST* p = 0.0050) and all markers combined (Fig. 7E, *SPAST*-Nos vs untreated controls p = 0.7792; *SPAST*-Nos vs untreated *SPAST* p < 0.0001).

However, noscapine treatment had a much smaller effect on nucleus and cell phase contrast morphology. The nucleus and cell phase contrast mean probability values of noscapine treated *SPAST* patient cells shifted away from untreated *SPAST* cell values and towards untreated control cell values. However, the noscapine treated *SPAST* patient cell values were not statistically significantly different from untreated *SPAST* patient or untreated control cell values. Nucleus (Fig. 7C, *SPAST*-Nos vs untreated controls p = 0.9570; *SPAST*-Nos vs untreated *SPAST* p = 0.1018) and cell phase contrast (Fig. 7D, *SPAST*-Nos vs untreated controls p = 0.9302; *SPAST*-Nos vs untreated *SPAST* p = 0.0568).

#### SPG7

The logistic regression model built while analysing control and *SPG7* patient samples (Fig. 4) was used to test if the morphology of noscapine treated *SPG7* patient cells are more similar to untreated control or untreated *SPG7* patient cells. We applied the model to test noscapine treated *SPG7* patient cells (16,379 cells from 5 individuals) and also to untreated control and *SPG7* patient cells presented above in the *SPG7* vs controls section (Fig. 4) for comparison.

**Figure 8:**
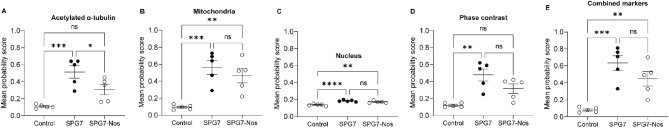
Logistic regression analysis of noscapine-treated *SPG7* patient cells. We compared noscapine treated *SPG7* patient cells (16,379 cells from 5 individuals) to untreated control and *SPG7* patient cells presented above in the *SPG7* vs controls section (Fig. 4) for comparison. Noscapine-treated patient cells had logistic regression probability values comparable to controls for acetylated α-tubulin (**A**). The markers mitochondria (**B**), nucleus (**C**), phase contrast (**D**) and all markers combined (**E**) were comparable to untreated SPG7 samples. Mean values were compared using one way ANOVA.

Noscapine treatment altered morphologies of all markers of *SPG7* patient cells to varying degrees (Fig. 8). The logistic regression probability mean values for all markers were significantly different between untreated control, untreated *SPG7* and noscapine treated *SPG7* patient groups (ANOVA analysis: acetylated α-tubulin p = 0.0007, mitochondria p = 0.0009, nucleus p < 0.0001, cell phase contrast p = 0.0015, markers combined p = 0.0004).

Noscapine treatment rescued *SPG7* patient cell acetylated α-tubulin morphology. Tukey’s multiple comparison test indicated that noscapine treated *SPG7* patient cells were statistically significantly different to untreated *SPG7* patient cells and similar to untreated control cells specifically for acetylated α-tubulin (Fig. 8A, *SPG7*-Nos vs untreated controls p = 0.0557; *SPG7*-Nos vs untreated *SPG7* p = 0.0486).

Noscapine treatment did not rescue *SPG7* patient cell mitochondria, nucleus, and cell phase contrast morphologies. There was no statistical significance observed between untreated *SPG7* and noscapine treated *SPG7* cells for mitochondria (Fig. 8B, *SPG7*-Nos vs untreated *SPAST* p = 0.5789), nucleus (Fig. 8C, *SPG7*-Nos vs untreated *SPG7* p = 0.1828), cell phase contrast (Fig. 8D, *SPG7*-Nos vs untreated *SPG7* p = 0.1111) and all markers combined (Fig. 8E, *SPG7* Nos vs untreated *SPG7* p = 0.1923).

## Discussion

In this study we show that automated image analysis and a machine learning-based method can distinguish between healthy control fibroblasts and those from two HSP genotypes (*SPAST* and *SPG7*). Moreover, the method distinguishes the genotypes from each other and demonstrates the reversal of genotype-associated cell morphologies after treatment with a low dose of the tubulin-binding drug, noscapine. We also show here the improved sensitivity gained by combining multiple cell features in a machine learning classification, compared to more commonly used methods of comparing individual cell features.

We present a range of novel findings. (A) *SPAST* and *SPG7* cells can be classified based on cell morphology with 100% sensitivity if all morphological features are combined in the analysis. Subsets of features are less sensitive in making the classifications. (B) Noscapine restored acetylated α-tubulin to control levels in *SPG7* cells, an unexpected finding because the mutation is in paraplegin, a mitochondrial associated protein^14^, raising the possibility of using noscapine as a treatment for these patients. Noscapine also restored acetylated α-tubulin to control levels in *SPAST* cells. This was expected from previous work^13^. In *SPAST* cells noscapine also restored mitochondrial, nucleus and phase contrast markers, demonstrating its effect more broadly on cell phenotype. C) Combining the cell morphology features or just the mitochondrial features in the machine learning models greatly improved the ability to detect the effect of mitochondrial inhibitors. Even the unlabelled cell features extracted from the phase contrast images distinguished between treated and untreated cells without overlap.

The power of cell morphological assays for cell classification may depend on the features chosen for analysis of cell components, i.e. which cell components are used. In this study we chose acetylated α-tubulin and mitochondrial markers based on known differences in the *SPAST* and *SPG7* cells^4,15^. For other diseases and genotypes, specific cell component markers could be chosen based on the disease pathology. Nonetheless, in this study when the subset of features extracted from the phase contrast images were used without contribution of the specific cell component markers the classification of the cell types was still very high. Unlabelled, phase contrast images would be the simplest way to classify cells and would be the cheapest and quickest method for high throughput screening of large compound libraries for drug discovery.

Genetic testing is one of the most commonly used diagnostic tests to diagnose patients in clinical practise and to recruit patients for clinical trials. On average, genetic testing can identify *SPAST* disease mutation in only 50% of clinically diagnosed cases. This makes it challenging to recruit sufficient patient numbers, particularly for a rare disease. Hence, there is a need for biomarkers allowing to identify patients that may benefit from a particular drug treatment. The method presented here can classify *SPAST* and *SPG7* patients with 100% specificity with the potential for predicting the genotype of individuals, although we did not test that here. The indication of the possibility is that the machine learning models used to discriminate *SPAST* and *SPG7* fibroblasts from controls were able to detect the effect of noscapine, classifying the treated cells as controls.

This is the first evaluation of cell morphology profiling for classifying HSP subtypes and the effect of a drug on those profiles. This machine learning-based cell morphology analysis is a generic method that can be applied to all neurodegenerative diseases for which fibroblasts can be obtained. Applications of single cell morphology include biomarkers for disease status, disease progression and their application in patient selection and treatment efficacy in clinical trials. Going forward, it will be interesting to evaluate larger patient groups with different disease severities, to test if cell morphology alone can indicate severity. For drug screening experiments when screening large numbers of compounds, this biomarker approach can be useful for primary screening, especially when using unlabelled cell phase contrast images. This can save money (antibodies are expensive) and time (imaging, instrument use and analysis) and improve efficiency (human errors with immunostaining protocols) especially. This can be followed by secondary screens on hit compounds that can evaluate the morphological features of specific cell components using component-specific labels. Morphometrics will provide new insights into biology of neurodegenerative disease and provide more genomically-precise ways to evaluate treatments for neurodegenerative diseases.

## Methods

### Ethics approval

Our study involving human cells was reviewed and approved by Human Research Ethics Committee affiliated to the Northern Sydney Local Health District, New South Wales government, Australia. The ethics committee reference number: RESP/15/314. All methods were carried out in accordance with relevant guidelines and regulations. The participants provided their written informed consent to participate in this study.

### Participants

HSP patients involved in this study were examined by Prof Carolyn Sue, an experienced movement disorder specialist. Disease mutation and related details of the patients and controls are listed in Table 1. Skin fibroblasts were obtained with written and informed consent of the participants.

**Table 1:** Mutation details of HSP patients with *SPG7* and *SPAST* mutations.

Serial ID	Cell line ID	Age at biopsy	Gender	Exon/intron	Nucleotide change	Protein change	Reference
**HSP patients with *SPAST* mutations**							
1	8076	47	F	3	c.583C>G	p.Leu195VaI	Vandebolan et al. (sibling of 08077)
2	6075 (or 08091)	35	M	8–9			Vandebolan et al
3	9006	24	M	17	c.1789A>G	p.Ser597GIy	Vandebolan et al
4	9007	42	F	17	c.1789A>G	p.S597G	Abretamsen et al. (referenced as H902)
5	6040	77	F	10	c.1291C>T	p.Arg431X	Vandebolan et al
6	9017	63	F	16			Vandebolan et al
7	9016	36	M	16			Vandebolan et al
8	9065	46	F	8–9			Vandebolan et al
9	4011	63	M	9	c.1196C>T	p.Ser399Leu	Vandebolan et al
10	9062	68	F	12	c.1466C>G	p.Pro489Arg	Vandebolan et al
**HSP patients with *SPG7* mutations**							
1	08/081	53	M	Exon11	c.1529C>T (het)	p.A510V	Wali et al. (patient ID: patient 3)
				Intron10	c.1449 + 1G>A (het)	–	
2	08/082	51	F	Exon11	c.1529C>T (het)	p.A510V	Same mutation as 08/081
				Intron10	c.1449 + 1G>A (het)	–	
3	04/009	44	F	Exon11	c.1529C>T (het)	p.Ala510V	Common to all our *SPG7* patients
				Exonl1	c.1745G>A	p.Gly582Asp	Pathogenic variant previously unreported
4	11/082	58	M	Exon11	c.1529C>T (het)	p.A510V	Wali et al. (patient ID: patient 2)
				Exon13	c.1727C>G (het)	p.S576W	
5	14/065	70	M	Exon11	c.1454_1462del (het)	p.Arg485_Glu487del	Wali et al. (patient ID: patient 1)
				Exon11	c.1529C>T (het)	p.A510V	
**Controls**							
1	10006	65	M				
2	10013	86	M				
3	10014	68	M				
4	10017	49	M				
5	10022	31	M				
6	10026	62	F				
7	10041	55	F				
8	11038	26	F				
9	11041	51	M				

Vandebona et al. (PMID: 23252998), Abrehamsen et al. (PMID: 23264559) and Wali et al. (PMID: 32973427).

### Cell seeding and immunostaining to identify cell components

Cells were seeded in 96 well plates (CellCarrier-96 Ultra Microplates, PerkinElmer). About 18,000 cells were seeded per well of a 96 well plate. Cells were immunostained with conjugated antibodies against acetylated α-tubulin (Santa Cruz, sc-23950) and TOM20 (ab210665, Abcam) to label stabilised microtubules and mitochondria, respectively. Immunostaining was performed using the fixation and permeabilization kit CytoFix and CytoPerm (554714, BD Biosciences) following this procedure. (a) Media from the 96 well plate was aspirated out and cells were washed twice with Dulbecco's phosphate-buffered saline (DPBS), (b) Cells were fixed using the CytoFix reagent for 25 min, followed by two DPBS washes, (c) Cells were permeabilised using the CytoPerm reagent for 30 min, (d) Cells were immunostained using the conjugated antibodies for 1 h, followed by two DPBS washes, (e) Cells were labelled with CellMask DeepRed dye (H32721, Invitrogen™) and Hoechst (Thermo Scientific™) for single cell identification and segmentation.

### Cell imaging

Images from five fluorescent channels were captured at 20 × magnification on the high throughput imaging system, Opera Phenix High-Content Screening System (PerkinElmer) excitation/emission wavelength: DAPI (375/456), Phase contrast (740/0), acetylated α-tubulin-GFP (488/522), TOM20-RFP (561/599) and CellMask-DeepRed (640/704). Five field of views were acquired per well. Duplicate wells were imaged per sample.

### Image processing and morphological feature extraction

The workflow for image processing and cell morphology feature extraction was performed using the Harmony High-Content Imaging and Analysis Software (version 4.1, Perkin Elmer). The cell nucleus and the cells were identified and segmented using the “Find Nuclei” and “Find cytoplasm” functions. Cellular morphological features related to size, shape, intensity, distribution pattern and texture intensity were measured for the cell (using phase contrast images), nuclei (Hoechst) and cell components microtubules (acetylated α-tubulin) and mitochondria (TOM20) using the functions “Calculate Intensity properties”, “Calculate morphology properties” and “Calculate texture properties”. Below is the detailed description of the advanced morphological features measured and analysed.

### Morphological feature interpretation

All morphological features measured are shown in Supplementary Fig. 1.

After selecting the cell cytoplasm, properties of cell features can be quantified, this includes fluorescence intensities within different cell regions (Supplementary Fig. 1A), basic morphological features (area, length, width, roundness), advanced morphological features (STAR properties), cell texture features (SER properties).

Symmetry, Threshold compactness, Axial, Radial (STAR) properties—(1) Symmetry: this involves a set of eight properties that characterize the symmetry of intensity distribution inside the cells. Properties are named “Symmetry XY”. X described intensity decay in the radial direction (0 or 1). Y reflects the number of nodal lines (similar to symmetry axis) (2 to 5) (Supplementary Fig. 1B). (2) Threshold compactness: A set of four properties describing how compact the brightest features inside the cell are. Supplementary Fig. 1C shows an object region with increasing compactness. (3) Axial: Characterize the cell axis ratio. This involves two properties quantify the length and length ratio of the two-principal axis of the objects (cells). Supplementary Fig. 1D shows principal axes of the nucleus. (4) Radial: Characterize the intensity distribution in radial direction. Radial Mean is the mean object radius based on the intensity values weighted by the distance from the mass center. Radial Relative Deviation characterizes the homogeneity of the fluorescence distribution (Fig. 1E). (5) Profile: Characterize the location of the intensity in cell regions with a weighted profile (Supplementary Fig. 1F). (6) Texture: Morphological properties of cells selected by filters such as spots (granularity) or valley (smooth filamentous objects) are calculated (Supplementary Fig. 1G).

### Binary logistic regression

We applied binary logistic regression analysis, a machine learning predictive analysis algorithm that uses the morphological features of cells as predictors. Cells belonging to two different groups are coded as 0 (Group 1) and 1 (Group 2). Based on the predictors, for each cell the analysis predicts a probability score. This score is between 0 and 1. This analysis is performed on thousands of cells per group. Using the single cell probability values, we plot a histogram for cells of the two groups and calculate the mean group probability scores. This is useful in comparing multiple patient and control samples. This analysis was performed using IBM SPSS 26 and GraphPad Prism 9.

### Machine learning based analysis of multiple morphological features to detect treatment effects of oligomycin/antimycin A

We treated skin fibroblasts from five healthy individuals with oligomycin (1.25 µM) and antimycin a (0.5 µM) (Sigma), inhibitors of the mitochondria respiratory chain, for 16 h and assessed cell and cell component morphology changes. 4370 untreated and treated cells were imaged. 31 morphological features were calculated per cell or cell component, this amounting to 541,880 morphological feature values (31 morphological parameters × 4 cell markers × 4370 cells). For logistic regression analysis, cells belonging to two different groups were coded as 0 (untreated) and 1 (treated).

### Machine learning based analysis of multiple morphological features to detect treatment effects of noscapine

We treated skin fibroblasts from *SPAST* patients and *SPG7* patients with noscapine at 10 µM for 24 h. The noscapine dosage used here is based on our previous results testing *SPAST* patient olfactory and cortical neurons-derived from induced pluripotent stem cells^16,17^. Cells were imaged and 124 morphological features were calculated (all markers combined). For logistic regression analysis, cells belonging to two different groups were coded as 0 (untreated) and 1 (treated).

### Data normalisation and reproducibility

Imaging experiments are subjected to day-to-day and batch-to-batch variations and it might result in wrong classification/bias in the logistic regression analysis.

We tested if the same samples imaged and analysed on different days effected cell morphologies and the resulting analysis. We tested the same four healthy control cell lines on three different days and tested if our logistic regression analysis of all markers combined (acetylated α-tubulin, mitochondria, nucleus and cell phase contrast) found any difference between these runs on different days. Logistic regression analysis of Run1 vs Run2, identified the same mean probability scores (0.48, Supplementary Fig. 2A) for all four samples in both Runs. Similarly, logistic regression analysis of Run1 vs Run3, identified the same mean probability scores (0.51, Supplementary Fig. 2B) for all four samples in both Runs. Although in both experiments i.e., Run1 vs Run2 and Run1 vs Run3, no difference group mean differences were seen, the mean values varied slightly (0.48 vs 0.51). This indicated a need for data normalisation. To address this, we normalised all our morphology values in all experiments to negative controls (healthy controls).

## Supplementary Information

Supplementary Information.
