# Supplementary material for: Single cell morphology distinguishes genotype and drug effect in Hereditary Spastic Paraplegia
Source: Sci Rep. 2021 Aug 17;11:16635. doi: 10.1038/s41598-021-95995-4 (PMC8371156; doi:10.1038/s41598-021-95995-4)
Supplement: Supplementary file 1 — Supplementary Information. [file 41598_2021_95995_MOESM1_ESM.docx]

**Supplementary file Single cell morphology distinguishes genotype and drug effect in Hereditary Spastic Paraplegia**


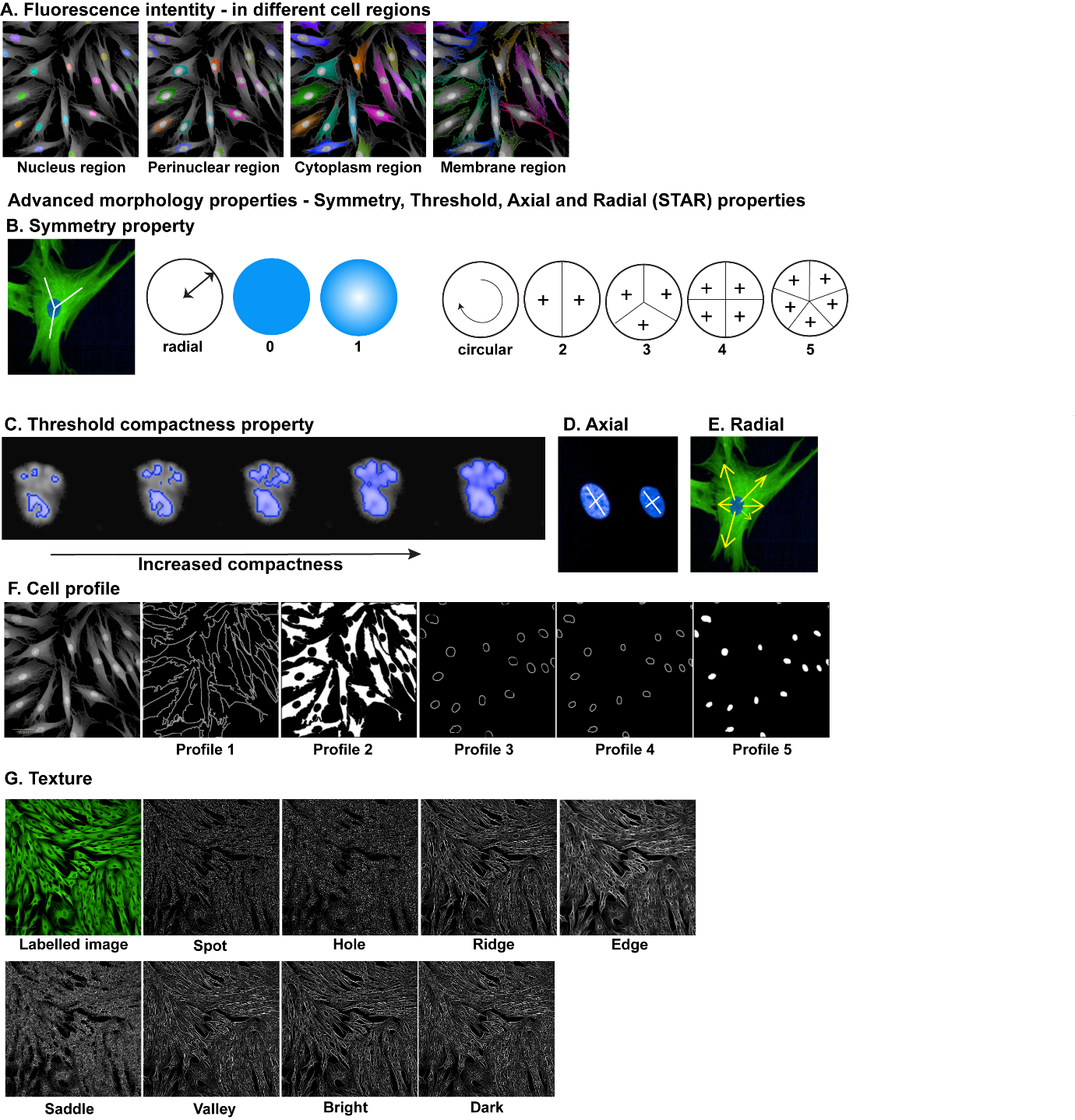


**Supplementary figure 1: Morphological features.** The morphological features of cell images were analysed and generated using Harmony High-Content Imaging and Analysis Software (version 4.1, Perkin Elmer). Morphological features include fluorescence intensities within different cell regions (**A**), basic morphological features (area, length, width, roundness), advanced morphological features - Symmetry, Threshold compactness, Axial, Radial (STAR) properties. (**B**) Symmetry: This involves a set of eight properties that characterize the symmetry of intensity distribution inside the cells. Properties are named “Symmetry XY”. X described intensity decay in the radial direction (0 or 1). Y reflects the number of nodal lines (similar to symmetry axis) (2 to 5) (Supplementary figure 1B). (**C**)Threshold compactness: A set of four properties describing how compact the brightest features inside the cell are. The image shows an object region with increasing compactness. There is no actual visual output of the threshold compactness measure from Harmony to help describe the feature. The threshold compactness image presented here is from Harmony image analysis guide, 7^th^ edition. (**D**) Axial: Characterize the cell axis ratio. This involves two properties that quantify the length and length ratio of the two-principal axis of the objects (cells). The image shows principal axes of the nucleus. (**E**) Radial: Characterize the intensity distribution in radial direction. Radial Mean is the mean object radius based on the intensity values weighted by the distance from the mass center. Radial Relative Deviation characterizes the homogeneity of the fluorescence distribution. (**F**) Profile: Characterize the location of the intensity in cell regions with a weighted profile. (**G**) Texture: Morphological properties of cells selected by filters such as spots (granularity) or valley (smooth filamentous objects) are calculated. 31 morphological features were calculated per cell (phase contrast image) or cell component (acetylated α-tubulin/ mitochondria/nucleus).


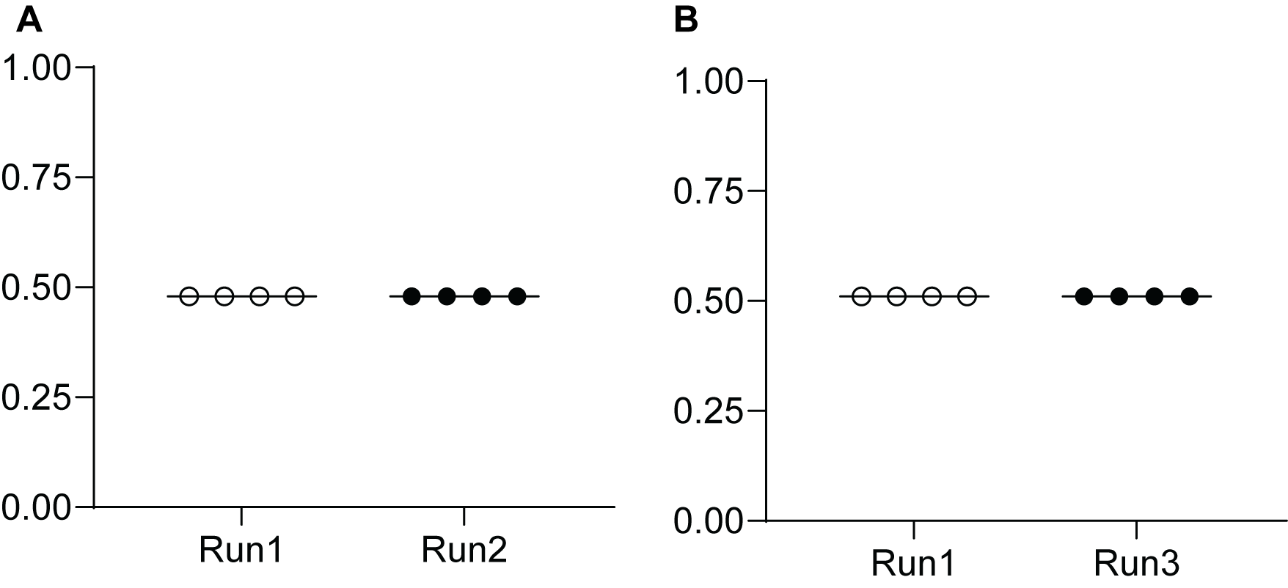


**Supplementary figure 2: Data normalisation and Reproducibility.** We tested if the same samples imaged and analysed on different days effected cell morphologies and the resulting analysis. We tested the same four healthy control cell lines on three different days and tested if our logistic regression analysis of all markers combined (acetylated α-tubulin, mitochondria, nucleus and cell phase contrast) found any difference between these runs on different days. (**A**) Logistic regression analysis of Run1 vs Run2, identified the same mean probability scores (0.48) for all four samples in both Runs. (**B**) Similarly, logistic regression analysis of Run1 vs Run3, identified the same mean probability scores (0.51) for all four samples in both Runs. Although in both experiments i.e., Run1 vs Run2 and Run1 vs Run3, no difference group mean differences were seen, the mean values varied slightly (0.48 vs 0.51). This indicated a need for data normalisation. To address this, we normalised all our morphology values in all experiments to negative controls (healthy controls). The mean values could not be compared using any statistical test as all mean values were same.
